# Characterisation of *Listeria monocytogenes* Isolates from Hunted Game and Game Meat from Finland

**DOI:** 10.3390/foods11223679

**Published:** 2022-11-17

**Authors:** Maria Fredriksson-Ahomaa, Mikaela Sauvala, Paula Kurittu, Viivi Heljanko, Annamari Heikinheimo, Peter Paulsen

**Affiliations:** 1 Department of Food Hygiene and Environmental Health, Faculty of Veterinary Medicine, University of Helsinki, 00014 Helsinki, Finland; 2 Microbiology Unit, Finnish Food Authority, 60100 Seinäjoki, Finland; 3 Unit of Food Hygiene and Technology, Institute of Food Safety, Food Technology and Veterinary Public Health, University of Veterinary Medicine, 1210 Vienna, Austria

**Keywords:** *Listeria monocytogenes*, game, whole genome sequencing, sequence type, virulence, antimicrobial resistance

## Abstract

*Listeria monocytogenes* is an important foodborne zoonotic bacterium. It is a heterogeneous species that can be classified into lineages, serogroups, clonal complexes, and sequence types. Only scarce information exists on the properties of *L. monocytogenes* from game and game meat. We characterised 75 *L. monocytogenes* isolates from various game sources found in Finland between 2012 and 2020. The genetic diversity, presence of virulence and antimicrobial genes were studied with whole genome sequencing. Most (89%) of the isolates belonged to phylogenetic lineage (Lin) II and serogroup (SG) IIa. SGs IVb (8%) and IIb (3%) of Lin I were sporadically identified. In total, 18 clonal complexes and 21 sequence types (STs) were obtained. The most frequent STs were ST451 (21%), ST585 (12%) and ST37 (11%) found in different sample types between 2012 and 2020. We observed 10 clusters, formed by closely related isolates with 0–10 allelic differences. Most (79%) of the virulence genes were found in all of the *L. monocytogenes* isolates. Only *fos*X and *lin* were found out of 46 antimicrobial resistance genes. Our results demonstrate that potentially virulent and antimicrobial-sensitive *L. monocytogenes* isolates associated with human listeriosis are commonly found in hunted game and game meat in Finland.

## 1. Introduction

*Listeria monocytogenes* has emerged over recent decades as an important foodborne pathogen responsible for numerous outbreaks [1]. *L. monocytogenes* is responsible for listeriosis, a disease affecting both humans and animals. Foodborne listeriosis typically causes a self-limited gastroenteritis among healthy people [2]. However, invasive infection leading to hospitalisation and even death may occur, especially among immunocompromised people [1]. Invasive listeriosis may also lead to abortion in pregnant women. The severity of listeriosis depends, inter alia, on the virulence of the bacterial strain [2]. Invasive listeriosis, in particular, requires antimicrobial treatment. Listeriosis had the highest proportion of hospitalised cases of all zoonoses in 2020 in the EU [3].

*L. monocytogenes* is a ubiquitous bacterium that can survive in a variety of environments and grow at low temperatures, e.g., in cold-stored foods [4]. Soil and decaying organic material are important sources of *L. monocytogenes*, and mammals and birds can spread this pathogen through faecal shedding [5]. *L. monocytogenes-*contaminated food is an important source attributed to human infections [6]. The consumption of contaminated food has been linked to both epidemic and sporadic listeriosis. Poor hygiene practices and inadequate sanitation procedures in the food processing industry can lead to listeriosis outbreaks [4,7].

*L. monocytogenes* has sporadically been found in game animals and on game carcasses [8,9,10,11]. Detection rates of *L. monocytogenes* in deer and wild boar faeces have been low, varying between 0 and 6% [12]. However, this pathogen is more frequently present in the tonsils than in faeces [12,13,14,15]. In Spain, *L. monocytogenes* was detected in 44% and 41% of deer and wild boar tonsils, respectively [12]. Recently, *L. monocytogenes* was detected in 5% of deer carcasses in Austria using an antigen test [16] and in 12% of deer carcasses in Finland using a polymerase chain reaction (PCR) [17]. This relatively high prevalence of *L. monocytogenes* on deer carcasses shows the importance of observing hygiene practices during hunting and slaughtering.

*L. monocytogenes* is a very heterogeneous species, which can be divided into at least 14 serotypes based on variation in the somatic (O) and flagellar (H) antigens [18,19]. Over 95% of the human and food strains are linked to four (1/2a, 1/2b, 1/2c, and 4b) serotypes. Genetically, *L. monocytogenes* can be divided into four phylogenetic lineages (Lin), six serogroups (SGs), multiple clonal complexes (CCs), and sequence types (STs) [2,5]. Most clinical strains found in humans belong to Lin I (SGs IIb and IVb) and II (SG IIa) [20], whereas food strains more frequently belong to SG IIa [21,22].

Whole genome sequencing (WGS)—an accurate method with a high resolution—is currently becoming the method of choice for characterising *L. monocytogenes* isolates [20]. It has emerged as a powerful tool for outbreak investigations and is increasingly also used for the surveillance and monitoring of listeriosis [23]. Investigating the diversity of *L. monocytogenes* isolates from game and game meat will provide valuable information on the significance of game in the meat production chain and in human infections.

Studies on the genetic relationships between *L. monocytogenes* isolates from game sources remain scarce. Game and game meat may play an important role in the *L. monocytogenes* infection cycle. The aim of our study was to use WGS to investigate the diversity and genetic relationships between *L. monocytogenes* isolates from hunted game and game meat in Finland. Furthermore, we studied the presence of important virulence and resistance genes obtained from the WGS data. 

## 2. Materials and Methods

### 2.1. Listeria Monocytogenes Isolates

*L. monocytogenes* has been detected in hunted game and game meat in Finland between 2012 and 2020, especially in deer and mallard meat (Table 1). We characterised a total of 75 *L. monocytogenes* isolates from various game sources in this study (Table 1). One isolate per positive sample from the earlier studies was characterised. The sampling plan and time frame differed between the earlier studies (Table 1). Moose, deer, and wild boar samples were collected from wild hunted animals and game bird samples from game birds that were farmed for hunting. Deer and mallard meat samples were collected from a small meat processing plant. *L. monocytogenes* isolates were found after PCR screening in our microbiological laboratory at the Department of Food Hygiene and Environmental Health of the Faculty of Veterinary Medicine, University of Helsinki, in Helsinki, Finland. PCR screening and isolation of the isolates have been described in earlier studies [17,24].

### 2.2. Whole Genome Sequencing (WGS)

DNA of *L. monocytogenes* isolates was purified from overnight enrichment at 37 °C in tryptic soya broth using PureLink Genomic DNA Mini Kit (Invitrogen, Carlsbaden, CA, USA) according to the manufacturer’s protocol. DNA quality was measured with a NanoDrop™ spectrophotometer (ThermoFisher Scientific, Waltham, MA, USA) and DNA quantity with a Qubit fluorometer (ThermoFisher Scientific). WGS was performed on the Illumina platform by CeGaT (Center for Genomics and Transcriptomics, Tübingen, Germany). Illumina DNA Prep library preparation kit and NovaSeq6000 were used to generate 100 bp paired end reads. The short raw reads were assembled de novo using a Unicycler v0.4.8 assembler available on the PATRIC 3.6.12 platform (https://www.patricbrc.org/app/Assembly, accessed on 11 November 2022). 

### 2.3. Characterisation of Listeria Monocytogenes Isolates

Species identification was confirmed in silico from the assemblies with KmerFinder v3.2 and SpeciesFinder v2.0 [26] available on the CGE (Center for Genomic Epidemiology) platform (http://www.genomicepidemiology.org/services/, accessed on 11 November 2022). In silico typing using 7-gene multi-locus sequence typing (MLST) [27] was performed on the CGE and BIGSdb-Lm (https://bigsdb.pasteur.fr/listeria/, accessed on 11 November 2022) platforms. STs obtained through the 7-gene MLST were grouped into CCs and phylogenetic Lin [27].

Assembled sequence data of 55 *L. monocytogenes* isolates were genotyped with core genome MLST (cgMLST) based on 1748 genes [28] using the open-source tool available on the BIGSdb-Lm platform. The nearest cgMLST profile (CT) from the database was recorded. Additionally, cgMLST targeting 1701 genes was performed using Ridom SeqSphere+ software v7.7.5 (Ridom GmbH, Muenster, Germany) [29] and the results were visualised with a minimum spanning tree (MST). Isolates forming a cluster (CL) displayed a maximum of 10 allelic differences from each other. The CLs were shaded in grey, and the number of allelic differences between the isolates was indicated on the connecting lines. Using the default parameters in the Ridom software, (1) STs, (2) PCR serogroups (SGs), (3) virulence genes and (4) antimicrobial resistance genes were also determined. Presence of the virulence genes was additionally studied with the VirulenceFinder 2.0 available on the CGE platform and on the Virulence Factor Database (VFDB) [30] (http://www.mgc.ac.cn/VFs/, accessed on 11 November 2022). In total, the presence of 33 virulence genes and 46 AMR genes among the 55 *L. monocytogenes* isolates was recorded.

## 3. Results

In total, 75 *L. monocytogenes* isolates from 75 hunted game and game meat samples—isolated in Finland between 2012 and 2020—were serotyped and characterised by seven-gene MLST (Table 2). Most (89%) of the isolates belonged to serotype 1/2a and were found in all sample types. Serotypes 4b and 2b were identified among 8% and 3% of the isolates, respectively. *L. monocytogenes* 4b was found on deer carcasses (n = 3), wild boar organs (n = 2) and in pheasant faeces (n = 1) (Table 2). *L. monocytogenes* 2b was only found in mallard faeces.

**Figure 1:**
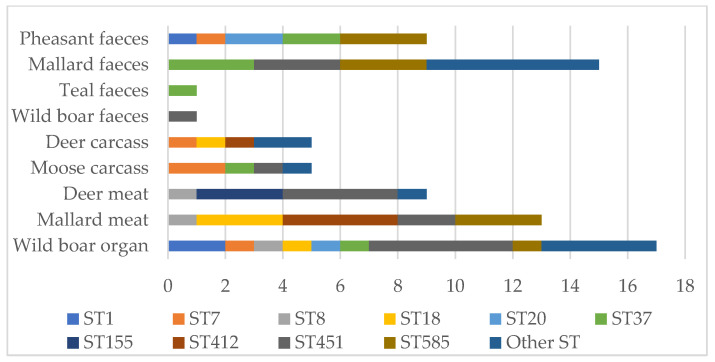
Sequence types (ST), which include at least three *Listeria monocytogenes* isolates, found in hunted game and game meat in Finland between 2012 and 2020.

Based on the seven-gene MLST, 75 *L. monocytogenes* isolates from hunted game and game meat samples (n = 75) were classified into Lin I and II, 18 CCs and 21 STs (Table 3). Most of the isolates (89%) belonged to Lin II, including all serotype 1/2a isolates. Lin I included isolates of serotypes 1/2b and 4b. ST451 (16/75) was the most frequent ST followed by ST585 (9/75) and ST37 (8/75). These STs were found from different sample types between 2012 and 2020 (Table 3). ST451 and ST37 have frequently been identified in human listeriosis during recent years in Finland (Table 3). In total, 8 out of 21 STs found in game have been identified in cases of human listeriosis in Finland between 2016 and 2021. Most (17/75) of the isolates from wild boars hunted in 2016 belonged to several (11/21) STs (Figure 1). *L. monocytogenes* isolates were also frequently found from mallard faeces (15/75) and mallard meat (13/75) (Table 2). These samples were contaminated with less (5/75) STs compared with wild boar organs.

**Figure 2:**
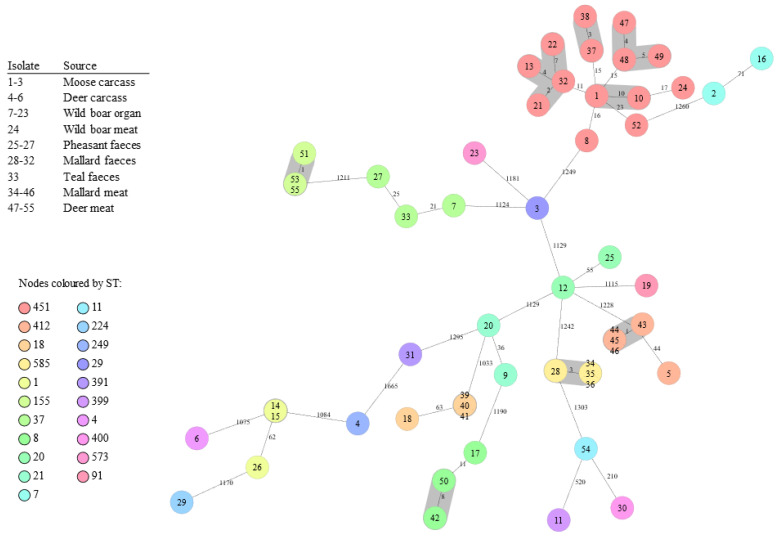
Minimum spanning tree of 55 *Listeria monocytogenes* isolates from hunted game and game meat in Finland during 2012–2020. The tree was calculated in Ridom SeqSphere+ with 1708 core genome multi-locus sequence typing (MLST) targets and 7-gene MLST targets (pairwise ignoring missing values, logarithmic scale). Nodes are coloured according to sequence type. Number of allelic differences between the isolates are indicated on the connecting lines. Clusters are shaded in grey and a cluster distance threshold of maximum10 was used according to Ruppitsch et al. [29].

A subset of 55 out of 75 *L. monocytogenes* isolates were characterised with cgMLST based on 1748 genes. In total, 35 CTs among 21 STs were obtained using the BIGSdb-Lm platform (Table 4). Overall, 10 CTs (CT5208, 11797, 20896, 20939, 25365, 26674, 26763, 28125, 28250, and 28251) included more than one *L. monocytogenes* isolate. In total, 10 CLs (CL1, 8, 18, 155, 412, 451a, 451b, 451c, 451d, and 585) formed by closely related genotypes were obtained with the cgMLST based on 1701 genes using the Ridom software (Table 4). Isolates belonging to the same CL showed an allelic difference between 0 and 10 (Figure 2). All CLs obtained by Ridom software had their own CT obtained from the BIGSSdb-Lm platform (Table 4). Most (9/10) of the CLs included isolates of SG IIa. CL1 included two undistinguishable isolates of SG IVb, both found from wild boar organs. Five CLs (CL1, 18, 155, 412 and 451c) included very closely related isolates, each with 0 to 5 allelic differences (Figure 2). Each of these CLs included *L. monocytogenes* isolates found from one source during the same year (Table 4). CL18 and CL412 were formed by isolates from mallard meat and CL155 and CL451c from deer meat. The other CLs (CL8, 451a, 451b, 451d and 585) were formed by closely related isolates (0 to 10 allele differences) found from different sources between 2012 and 2019 (Table 4).

We studied the presence of 33 virulence genes available in the Ridom software [34] among the 55 *L. monocytogenes* isolates. Most (26/33) of the genes were detected in all isolates. Seven virulence genes (*act*, *ami*, *aut*, *inl*F, *inl*J, *lap*B and *vip*) were not present in all isolates. We designed 12 virulence profiles (VPs) based on these genes (Table 5). All virulence genes (VP0) were detected in 12 (22%) *L. monocytogenes* isolates, all belonging to SG IIa. The most frequently missing genes were *ami* and *vip*, which were missing in 42% and 38% of *L. monocytogenes* isolates, respectively. The VP did not correlate with ST, but isolates belonging to the same cluster mostly (66%) had the same VP (Table 4). 

We studied the presence of 46 AMR genes available in the Ridom software. Only the *fos*X and *lin* genes were detected in all 55 *L. monocytogenes* isolates.

## 4. Discussion

*L. monocytogenes* is a common finding in hunted game and game meat in Finland. Most of the *L. monocytogenes* isolates originating from game belonged to serotype 1/2a (SG IIa, Lin II) but serotype 4b (SG IVb, Lin I) was also found. *L. monocytogenes* strains belonging to SG IIa/Lin II and SG IVb/Lin I are responsible for the largest share of listeriosis [20,35,36]. However, SG IVb is more frequently associated with human diseases and outbreaks than SG IIa, which is more often identified among isolates found in animal, environmental and food samples [6,18,21]. Recently, *L. monocytogenes* IIa and IVb were found in deer and wild boar tonsils in Spain [12]. Serotyping and serogrouping provide useful information about *L. monocytogenes* isolates found in epidemiological studies, surveys and during monitoring.

Very little is known about the genetic diversity of *L. monocytogenes* isolates from game origin [12]. We found several CCs and STs in hunted game and game meat from Finland showing a large genetic diversity among the *L. monocytogenes* isolates studied. This was expected because *L. monocytogenes* isolates were found from various sources and locations during a ten-year period [17,24,25]. All CCs identified among our hunted game and game meat isolates from Finland have recently been identified among various environmental and animal sources in Europe [5]. In our data, the most common CC was CC11 (25%), which included three STs: ST11, ST400 and ST451. CC11 is also a prevalent clonal type found in Europe [23,31]. Most (67%) of the STs found in our study have also been found in Europe from various sources [23,31,32]. Several (7/21) STs found in game in our study have been associated with human listeriosis in Finland (https://thl.fi/en/web/infectious-diseases-and-vaccinations accessed on 11 November 2022). ST451 (21%) was the most frequently found ST in our study, as it was found in different sample types between 2012 and 2020. This type has also been reported in human listeriosis in Finland yearly between 2017 and 2021. ST451 is a common universal ST found in humans, animals, foods, and the environment in Europe [23,31,32,37]. To obtain more accurate information about the link between human and game isolates, STs based on the core or whole genome (cgMLST or wgMLST) should be used instead of seven-gene MLST.

Wild boar organs were contaminated with several *L. monocytogenes* isolates of different STs. This is very understandable because the isolates originated from wild boars hunted in various geographical locations in Finland [25]. Fewer STs were found in isolates from deer and mallard meat than from wild boar. Deer and mallard meat were processed in one meat processing plant each, which may explain the limited genetic diversity among the meat isolates. Interestingly, only 4 STs were identified among 13 *L. monocytogenes* SG IIa isolates from mallard faeces. The hunted mallards were reared and fed in a natural pond before being hunted, which could be a common contamination source for the mallards [24]. *L. monocytogenes* is relatively commonly found in various environments, and *L. monocytogenes*-contaminated soil and water are therefore important *L. monocytogenes* sources [5,38]. ST18, ST20, ST37, ST91 and ST451, identified among our game isolates, are reportedly common STs among isolates from environmental samples in Finland [37] and Latvia [31].

We identified some CLs of *L. monocytogenes* isolates with 0 to 10 allelic differences among the hunted game and game meat isolates using cgMLST, which is the method capable of differentiating related strains from unrelated ones [39]. Very closely related isolates, with a maximum of five allelic differences, were found in five CLs, and they originated from the same source and year, which could explain the high genetic similarity and may indicate a common source of contamination. Three very closely related *L. monocytogenes* isolates—forming CL18—were isolated from mallard meat originated from various mallards sampled on the same day in the same game meat processing plant, indicating a cross-contamination during processing. In CL412, four very closely related isolates from mallards were sampled on two different days in the same plant, indicating a plant contamination possibly due to inadequate cleaning. Deer meat isolates also formed two clusters—CL155 and CL451c—both with three very closely related isolates. The isolates in CL155 and CL451c were from deer meat samples cut on different days in the same plant. Cross-contamination during meat cutting occurs easily if working hygiene is poor. *L. monocytogenes* can easily persist in the plant, and thorough cleaning of the meat processing plant after each working day is therefore very important. 

*L. monocytogenes* has shown heterogeneity in its virulence [35,40]. Virulence factors are essential for adapting *L. monocytogenes* to spread optimally within the environment [35]. The virulence of *L. monocytogenes* is encoded by a wide range of virulence genes [2]. In our study, most (79%) of the 33 studied virulence genes were present in all 55 *L. monocytogenes* isolates of game origin in Finland. The *act*A gene located on the *Listeria* pathogenicity island (LIPI-1) was missing in only one isolate (a deer carcass isolate). LIP-I is composed of important virulence genes (including *act*A, *hly, mpl*, *plc*A, *plc*B, *prf*A and *orf*X) and is necessary for intracellular survival and spread from cell to cell [35]. LPI-1 is typically present in all *L. monocytogenes* strains [2,41]. This *actA*-negative deer carcass isolate (with VP3b, SG IIa and ST412) also missed the *lap*B (coding for an adhesion protein) and *vip* (coding for an invasion protein) genes, indicating a reduced virulence in this isolate. The most frequently missing virulence gene *ami*, which is coding an autolysin protein for adherence, was not found in 42% of the isolates. However, the meaning of *ami* in the virulence remains unclear. The invasion gene *aut* was missing only in the isolates belonging to SG IVb. All SG IVb isolates (with ST1, ST4 and ST249) were *aut*-negative. The three SG IVb isolates belonging to ST1 were also *inl*J-negative. The *aut* gene codes for an autolysin protein needed for invasion and the *inl*J for an internalin protein needed for adherence [35]. How the absence of these two genes affects the virulence of *L. monocytogenes* IVb isolates needs to be further studied. Typically, ST1 (CC1) and ST4 (CC4) have been associated with clinical cases more often than other STs [35,41].

AMR is a serious public health issue due to increasing resistance. There is also a trend of increasing AMR among *L. monocytogenes* strains of animal and food origin. Resistance, e.g., to penicillin, ampicillin, gentamycin, streptomycin, tetracycline, and trimethoprim-sulfamethoxazole has been reported [42,43]. However, in our study, only the *fos*X and *lin* genes were detected. These two genes were present in all 55 *L. monocytogenes* isolates. Earlier studies have shown *fos*X and *lin* to be present in nearly all *L. monocytogenes* isolates [42]. This can be explained by native resistance to fosfomycin and lincosamides reported in *L. monocytogenes* strains [34,43]. Our results indicate that *L. monocytogenes* of game and game meat origin found in Finland are so far sensitive to antimicrobials. One explanation may be that hunted game in Finland have no access to feed contaminated with resistant *L. monocytogenes* strains.

## 5. Conclusions

In this study, we analysed the sequence data of *L. monocytogenes* isolates of game origin using tools available on open-source platforms and Ridom software. Our study demonstrates that game meat is contaminated with various STs associated with human listeriosis. All *L. monocytogenes* isolates were potentially pathogenic, carrying most important virulence genes. No acquired AMR genes were found, indicating that all isolates were sensitive to most of the important antimicrobials used to treat listeriosis. Some of the isolates from mallard and deer meat belonged to CLs that were formed by very closely related isolates, indicating common contamination sources. Contaminated game meat may pose a public health problem, and game meat should therefore be handled and stored correctly.

## Figures and Tables

**Table 1:** Isolation rates of *Listeria monocytogenes* from hunted game and game meat in Finland between 2012 and 2020.

Scheme 2012	Sampling Year	Number of Samples	Positive Samples		Reference
Moose carcass	2012–2014	100	5	(5%)	[17]
Deer carcass	2013–2015	100	5	(5%)	[17]
Deer meat	2019–2020	50	9	(18%)	Not published
Wild boar organ	2016	130	40	(31%)	[25]
Pheasant faeces	2013–2014	101	9	(9%)	[24]
Teal faeces	2013	30	1	(3%)	[24]
Mallard faeces	2013–2014	110	15	(14%)	[24]
Mallard meat	2016	100	13	(13%)	[24]

**Table 2:** Serotypes and sequence type (STs) using 7-gene multi-locus sequence typing (MLST) of 75 *Listeria monocytogenes* isolates obtained from hunted game and game meat in Finland between 2012 and 2022.

Source	Isolation Year	Number of Isolates	Serotype	MLST
Moose carcass	2012–2013	5	1/2a	ST7, 29, 37, 451
Deer carcass	2013–2014	5	1/2a (2), 4b (3)	ST4, 18, 315, 412
Deer meat	2019	9	1/2a	ST8, 11, 155
Mallard faeces	2013–2014	15	1/2a (13), 1/2b (2)	ST11, 37, 224, 391, 585
Mallard meat	2016	13	1/2a	ST8, 18, 412, 451, 585
Teal faeces	2013	1	1/2a	ST37
Pheasant faeces	2013–2014	9	1/2a (8), 4b (1)	ST1, 7, 20, 37, 585
Wild boar faeces	2020	1	1/2a	ST451
Wild boar organ	2016	17	1/2a (15), 4b (2)	ST1, 7, 8, 18, 20, 21, 37, 91, 399, 451, 573

**Table 3:** Characteristics of 75 *Listeria monocytogenes* isolates from hunted game and game meat from Finland between 2012 and 2020.

MLST	Finland ^b^	Clonal Complex	Lineage	Serotype	Number of Isolates	Source (Isolation Year)
**ST1** ^a^	2016, 2017	CC1	I	4b	3	Pheasant faeces (2013), Wild boar organ (2016)
**ST4**		CC4	I	4b	1	Deer carcass (2014)
**ST7**	2018–2021	CC7	II	1/2a	4	Moose carcass (2012), Pheasant faeces (2013), Wild boar organ (2016)
**ST8**	2017–2018, 2020–2021	CC8	II	1/2a	3	Mallard meat (2016), Wild boar organ (2016), Deer meat (2019)
ST11		CC11	II	1/2a	1	Deer meat (2019)
**ST18**	2016	CC18	II	1/2a	5	Deer carcass (2013), Wild boar organ (2016), Mallard meat (2019)
**ST20**		CC20	II	1/2a	3	Pheasant faeces (2013), Wild boar organ (2016)
**ST21**		CC21	II	1/2a	2	Wild boar organ (2016)
**ST29**		CC29	II	1/2a	1	Moose carcass (2013)
**ST37**	2016, 2018–2020	CC37	II	1/2a	8	Moose carcass (2013), Teal faeces (2013), Mallard faeces (2013–2014), Pheasant faeces (2013–2014) Wild boar organ (2016)
**ST91**	2021	CC14	II	1/2a	1	Wild boar organ (2019)
**ST155**	2020	CC155	II	1/2a	3	Deer meat (2019)
**ST224**		CC224	I	1/2b	2	Mallard faeces (2013)
ST249		CC315	I	4b	2	Deer carcass (2013)
ST391		CC89	II	1/2a	2	Mallard faeces (2013)
**ST399**		CC14	II	1/2a	1	Wild boar organ (2016)
ST400		CC11	II	1/2a	2	Mallard faeces (2013)
ST412		CC412	II	1/2a	5	Deer carcass (2013), Mallard meat (2019)
**ST451**	2017–2021	CC11	II	1/2a	16	Moose carcass (2012), Mallard faeces (2013–2014), Mallard meat (2016), Wild boar organ (2016), Deer meat (2019), Wild boar faeces (2020)
**ST573**		CC573	II	1/2a	1	Wild boar organ (2016)
ST585		ST585	II	1/2a	9	Pheasant faeces (2013–2014), Mallard faeces (2013–2014), Mallard meat (2016)

^a^ Sequence types in bold have recently been published in other European countries [31,32,33]. ^b^ Reporting year of the most common sequence types found in human listeriosis in Finland during 2016–2021.

**Table 4:** Distribution of different serogroups (SGs), MLST (STs) and cgMLST (CTs) profiles, clusters (CLs) and virulence profiles (VPs) among 55 *Listeria monocytogenes* isolates from hunted game and game meat between 2012 and 2020 in Finland.

ST ^a^	SG	CT ^b^	CL ^c^	VP ^d^	No.	Source		Year
1	IVb	1430		2c	1	Pheasant	Faeces	2013
1	IVb	25365	1	2c	2	Wild boar	Organ	2016
4	IVb	27292		1d	1	Deer	Carcass	2014
7	IIa	21218		2a	1	Wild boar	Organ	2016
7	IIa	22874		1e	1	Moose	Carcass	2012
8	IIa	19030		2a	1	Wild boar	Organ	2016
8	IIa	28125	8	1e	2	Mallard, Deer	Meat	2016, 2019
11	IIa	30149		1b	1	Deer	Meat	2019
18	IIa	26674	18	1a	3	Mallard	Meat	2016
18	IIa	28197		1a	1	Wild boar	Organ	2016
20	IIa	21358		1a	1	Wild boar	Organ	2016
20	IIa	26681		0	1	Pheasant	Faeces	2013
21	IIa	9841		2a	1	Wild boar	Organ	2016
21	IIa	25363		2a	1	Wild boar	Organ	2016
29	IIa	23920		1e	1	Moose	Carcass	2013
37	IIa	22893		1e	1	Teal	Faeces	2013
37	IIa	28230		1e	1	Pheasant	Faeces	2014
37	IIa	30787		2a	1	Wild boar	Organ	2016
91	IIa	20232		1a	1	Wild boar	Organ	2016
155	IIa	26763	155	0	3	Deer	Meat	2019
224	IIb	8887		1c	1	Mallard	Faeces	2013
249	IVb	20958		1d	1	Deer	Carcass	2013
391	IIa	29935		0	1	Mallard	Meat	2013
399	IIa	22031		2b	1	Wild boar	Organ	2016
400	IIa	28173		1b	1	Mallard	Meat	2013
412	IIa	8287		3b	1	Deer	Carcass	2013
412	IIa	20896	412	2d	4	Mallard	Meat	2016
451	IIa	24184		0	1	Deer	Meat	2019
451	IIa	11793		0	1	Wild boar	Faeces	2020
451	IIa	5208	451a	1a	3	Mallard, wild boar	Meat, organ	2016
451	IIa	11797	451b	0,1a	2	Moose, wild boar	Carcass, organ	2012, 2016
451	IIa	20939	451c	0	3	Deer	Meat	2019
451	IIa	28250	451d	0,1a	4	Mallard, wild boar	Faeces, organ	2014, 2016
573	IIa	1569		2a	1	Wild boar	Faeces	2016
585	IIa	28251	585	1e,2a,3a	4	Mallard	Faeces, meat	2013, 2016

^a^ ST based on 7-gene MLST using the BIGSdb-Lm platform and Ridom software. ^b^ Nearest CT based on cgMLST (1748 target genes) using the BIGSdb-Lm platform. ^c^ CL based cgMLST (1708 target genes) using the Ridom software. ^d^ VPs using the Ridom software and CGE platform.

**Table 5:** Virulence profiles detected among 55 *Listeria monocytogenes* isolates.

Virulence Profile	Number of Isolates	Sequence Types	Missing Virulence Genes (=1)						
			*act*	*ami*	*aut*	*inl*F	*inl*J	*lap*B	*vip*
VP0	12	ST20,155,391,451	0	0	0	0	0	0	0
VP1a	13	ST18,20,91,451	0	1	0	0	0	0	0
VP1b	2	ST11,400	0	0	0	1	0	0	0
VP1c	1	ST224	0	0	0	0	1	0	0
VP1d	2	**ST4,249 ^a^**	0	0	1	0	0	0	0
VP1e	7	ST7,8,29,37, 585	0	0	0	0	0	0	1
VP2a	7	ST7,8,21,37,573,585	0	1	0	0	0	0	1
VP2b	1	ST399	0	1	0	1	0	0	0
VP2c	3	**ST1**	0	0	1	0	1	0	0
VP2d	4	ST412	0	0	0	0	0	1	1
VP3a	2	ST585	0	1	0	0	1	0	1
VP3b	1	ST412	1	0	0	0	0	1	1

^a^ Isolates with sequence types in bold belong to serogroup IVb.

## Data Availability

Data are contained within the article.
